# GLP-1–targeted therapies for alcohol and other substance use disorders: a new era on the horizon?

**DOI:** 10.1172/JCI211153

**Published:** 2026-09-01

**Authors:** Lorenzo Leggio, W. Kyle Simmons

**Affiliations:** 1 Clinical Psychoneuroendocrinology and Neuropsychopharmacology Section, Translational Addiction Medicine Branch, National Institute on Drug Abuse Intramural Research Program and National Institute on Alcohol Abuse and Alcoholism Division of Intramural Clinical and Biological Research, NIH, Baltimore, Maryland, USA.; 2 Center for Alcohol and Addiction Studies, Department of Behavioral and Social Sciences, Brown University, Providence, Rhode Island, USA.; 3 Division of Addiction Medicine, Department of Medicine, School of Medicine, Johns Hopkins University, Baltimore, Maryland, USA.; 4 Department of Neuroscience, Georgetown University Medical Center, Washington, DC, USA.; 5 Department of Pharmacology and Physiology and OSU Biomedical Imaging Center, Oklahoma State University Center for Health Sciences, Tulsa, Oklahoma, USA.

Alcohol and other substance use disorders (SUDs) are chronic medical conditions associated with substantial medical, psychological, socioeconomic, and societal burden. The DSM-5 diagnosis of a SUD constitutes the presence of two or more of 11 symptoms over the past year, resulting in substantial clinical heterogeneity, with more than 2,000 symptom combinations possible. SUD includes three levels of severity: mild (2–3 symptoms), moderate (4–5 symptoms), and severe (6+ symptoms). The International Classification of Diseases system retains two diagnoses, harmful use (lower severity) and substance dependence (higher severity). Despite the availability of psychosocial, behavioral, and pharmacological treatments, SUDs remain leading causes of mortality and morbidity. FDA-approved medications exist for alcohol, opioid, and tobacco use disorders, but their numbers are few, and not all patients respond to these medications, underscoring the urgent need for developing additional effective medications.

Interestingly, a growing body of preclinical literature, coupled with human research (e.g., neuroimaging as well as epidemiological and behavioral studies), supports commonalities between substance use disorders (SUDs) and obesity (1–3). The neurocircuitry, pathways, and mechanisms regulating alcohol and other addictive drug seeking and use overlap greatly with those regulating food seeking and consumption (1–3). These shared mechanisms span both brain-specific and periphery-brain pathways, including neuroendocrine gut-brain systems such as glucagon-like peptide-1 (GLP-1) signaling (4), as detailed in this Viewpoint.

## GLP-1–targeted therapies and addiction

Growing literature supports the potential role of GLP-1 receptor agonists (GLP-1RAs) such as semaglutide in treatment of SUDs. Across multiple animal models and species, GLP-1RAs reduce drug intake and other translationally relevant drug-related outcomes, including conditioned place preference and reinstatement (5). In parallel, a plethora of electronic health record–based (EHR-based) pharmacoepidemiological studies, some employing target trial emulation approaches, have linked GLP-1RA prescription receipt with improved outcomes in SUDs, including longer remissions and reduced alcohol/drug intake, hospitalization, overdose, and mortality (6). Perhaps most critically, a few recent randomized controlled trials (RCTs) provide support for the role of GLP-1RAs in treating SUDs (for review, see Farokhnia and Leggio, 2026) (5). Specifically, although an initial RCT of once-weekly exenatide in people with alcohol use disorder (AUD) found no difference between the active drug and placebo on the primary alcohol drinking outcomes in the overall sample (7), three more recent RCTs evaluating either weekly subcutaneous semaglutide (8, 9) or daily oral semaglutide (10) provide converging evidence that semaglutide reduces alcohol consumption in people with AUD. The RCT literature on people with tobacco use disorder (TUD) is more mixed. Compared with placebo, once-weekly exenatide led to a significantly higher smoking abstinence rate and lower craving scores (11), whereas once-weekly dulaglutide was not superior to placebo with respect to either smoking abstinence or craving (12). A more recent RCT in people with TUD found that once-weekly semaglutide reduced nicotine craving but had no effect on laboratory smoking resistance or cigarettes smoked per day (13). All 3 RCTs reported beneficial effects on weight (11–13), including reduced postcessation weight gain (11, 12). Finally, a 3- to 5-week pilot RCT conducted in a residential opioid use disorder (OUD) treatment program showed that the once daily liraglutide was safe and led to a reduction in opioid craving in people with OUD (unpublished observations; for further information, see clinicaltrials.gov, NCT04199728) (14).

**Figure 1:**
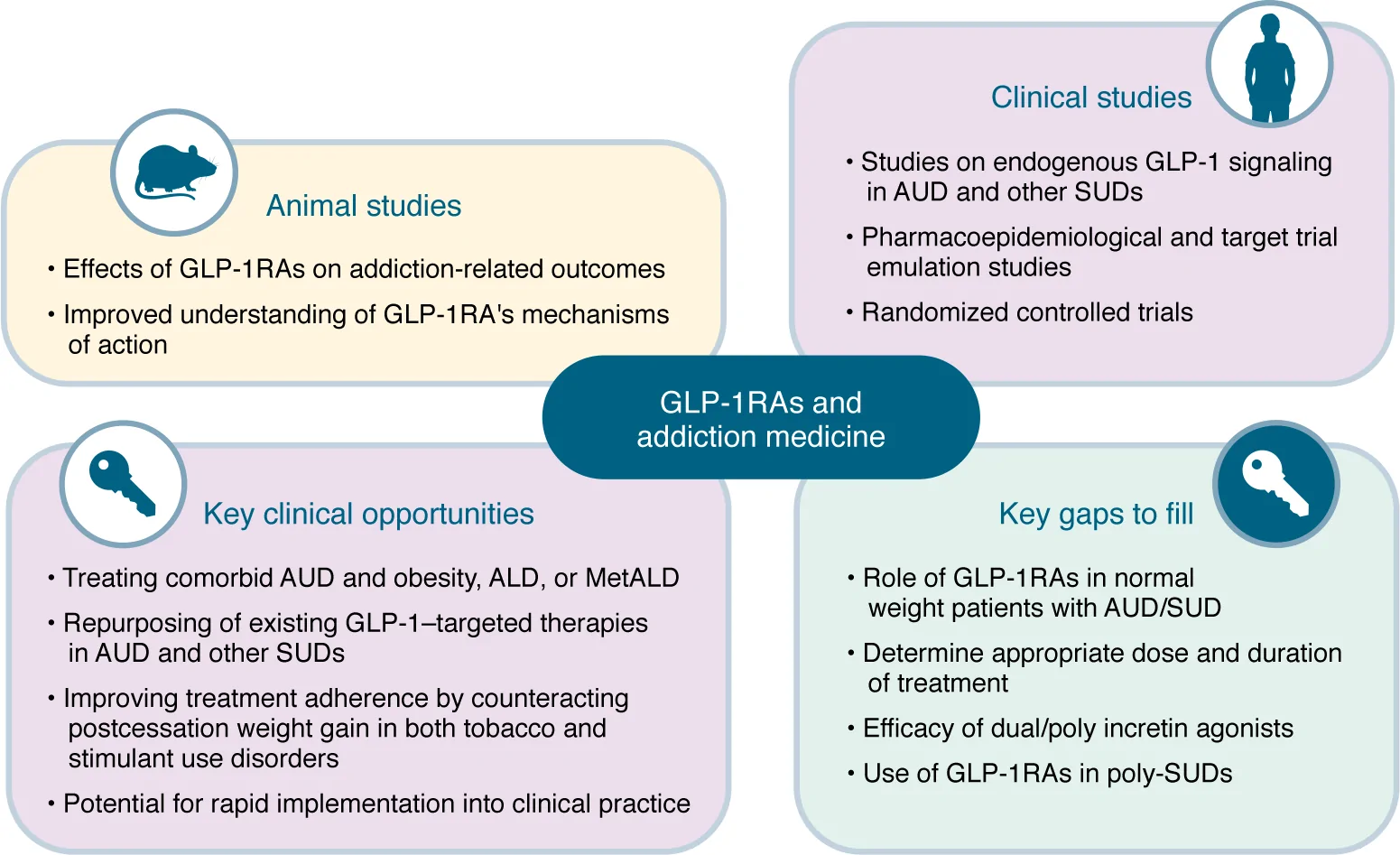
The emerging translational landscape of GLP-1–targeted therapies in AUD and other SUDs. ALD, alcohol-associated liver disease; AUD, alcohol use disorder; GLP-1RA, GLP-1 receptor agonist; MetALD, metabolic dysfunction–associated alcohol-related liver disease; SUD, substance use disorder.

Collectively, these findings have generated substantial enthusiasm for the potentially transformative role of GLP-1–targeted therapies in addiction medicine. Given the widespread use of GLP-1RAs and prevalence of SUDs, should their effectiveness be confirmed, this class of medications may already represent the most widely used pharmacotherapy for addictive behaviors ever developed. This Viewpoint focuses on emerging opportunities and key unanswered questions (Figure 1).

## New opportunities and unprecedented trends

The expanded landscape of GLP-1RAs, ranging from cardiometabolic and liver diseases to neurodegenerative and neuropsychiatric disorders, positions this class of medications uniquely within addiction medicine. Beyond improving SUD-related outcomes (e.g., abstinence, reduction in use), GLP-1RAs may simultaneously address the many comorbidities common in people with SUDs, including obesity, sleep disorders, liver diseases, cardiovascular diseases, and neuropsychiatric disorders. As an example, alcohol is a major contributor to liver diseases, including alcohol-associated liver disease, and metabolic dysfunction and alcohol-associated liver disease. Thus, GLP-1–targeted therapies may represent a much-needed natural bridge between addiction medicine and hepatology (15). Importantly, concerns about unforeseen safety issues during real-world implementation of GLP-1RAs to treat SUDs may be somewhat allayed for at least three reasons. First, these medications are already widely prescribed and, given the high prevalence of SUDs in the general population, many people affected by an SUD are likely taking them for type 2 diabetes and/or obesity. Second, their expanding clinical use may facilitate simultaneous treatment of SUDs and related medical and mental health comorbidities. Third, the available RCT data suggest that GLP-1RA safety profiles in populations with SUDs are comparable to those observed in GLP-1RA RCTs for diabetes and obesity (7–14). If effectiveness for SUDs is confirmed, GLP-1RAs represent an exciting and unprecedented example of drug repurposing and implementation science.

Scientific momentum has accelerated rapidly in studying GLP-1–targeted therapies as promising treatments for SUDs and comorbid conditions, with growing investment from NIH (via its intramural research program, extramural grants, contract-based RCTs), academia, pharmaceutical companies, and other private/philanthropic entities. In tandem, patients taking GLP-1RAs for diabetes or obesity are anecdotally reporting reductions in drinking and/or cigarette smoking and other drug use, which has attracted widespread media and public attention. The net result is an unprecedented opportunity for GLP-1RAs to transform addiction medicine, similar to how selective serotonin reuptake inhibitors advanced treatment of depression and other mood disorders a few decades ago. Longstanding challenges in the field include not only the limited number of approved medications, but also their low uptake by the patient population (16), which is driven by numerous, potentially synergistic, factors: stigma, limited awareness among those affected that effective treatments exist, gaps in clinician education and training, and insufficient structural support within healthcare institutions. The leitmotiv accompanying all these factors is the profound stigma of addiction. GLP-1RAs may help to dismantle this stigma. Public discussion of GLP-1–targeted therapies is already increasing awareness of SUDs as treatable chronic medical conditions and highlighting the existence of both GLP-1RA and non–GLP-1–based pharmacologic treatments alike, including those already approved by the FDA. Moreover, because GLP-1RAs are already widely accepted in clinical practice for type 2 diabetes and obesity, both patients and clinicians may feel more comfortable using them than traditional SUD medications. If proven effective, GLP-1–targeted therapies could substantially increase engagement with evidence-based SUD treatment, expand prescribing beyond specialty settings, and contribute broadly to the destigmatization of addiction and SUDs.

Notably, the exact mechanisms by which GLP-1–targeted therapies may help people with SUDs are still not fully understood. The best-supported mechanism is the ability of GLP-1RAs to reduce the hedonic value not only of food but also of addictive substances by directly and indirectly reducing dopamine transmission in mesolimbic areas related to reward processing (4). More broadly, GLP-1RAs seem to act on neural pathways involved in processing reward, motivation, and elements of wanting and liking that are often implicated as contributing factors in SUDs (4). Additionally, GLP-1 acts on brain areas involved in stress response and emotion regulation (17), which are also critically implicated in the etiology, development, and maintenance of SUDs. This suggests that stress-related and affective mechanisms may also play a role in how GLP-1RAs affect SUD-related outcomes (4, 5). Other mechanisms may include those related to satiety, interoception, taste and smell, neuroprotection, and neuroinflammation (5).

## Gaps to fill

Many important open questions remain. Chief among them are the optimal dose and duration of GLP-1RA therapy for SUDs. For example, low-dose semaglutide (up to 0.5 mg/week) reduced alcohol drinking in nontreatment seeking people with AUD, especially in those without obesity (BMI <30 Kg/m^2^) (8), while a 5-month RCT demonstrated efficacy of a higher dose of semaglutide (up to 2.4 mg/week) in people with AUD and obesity (9). People taking GLP-1RAs for weight loss may experience a pronounced rebound in appetite after discontinuing treatment. This well-known observation raises the question of whether a similar phenomenon may occur in people with SUDs who are treated with GLP-1RAs. Although long-term follow-up studies will be necessary to address this question, two important concepts should be considered. First, a return to pretreatment alcohol or drug use is commonly observed in clinical practice among patients who discontinue treatment, regardless of the specific pharmacological and/or behavioral approach used (18, 19). Second, SUDs are chronic medical disorders that require ongoing follow-up and long-term care. For example, if proven effective, GLP-1RAs could be used for a defined period as a bridge to transition patients to behavioral and/or psychosocial interventions for long-term maintenance. Furthermore, just like any other treatment for virtually any medical condition, if proven to be effective for SUDs, GLP-1RAs will not work equally for everyone. Genetic, metabolic, and other biological and/or environmental factors may influence safety, tolerability, and efficacy of GLP-1–targeted therapies. These determinants remain poorly understood, however.

The emergence of oral formulations and GLP-1 dual or poly-agonists, which emulate other incretin molecules, such as glucose-dependent insulinotropic polypeptide and glucagon, also represent important areas to investigate in the context of SUDs (5). These poly-agonists have the potential for providing stronger effects, but their safety and tolerability in people with SUDs require careful investigation. These poly-agonist approaches also raise mechanistic questions that require continued integration of basic neuroscience and clinical trials.

GLP-1 signaling influences not only appetitive and consummatory behaviors, but also reward processing, stress and mood, neuroinflammation, aversion, and interoception (20). Thus, further investigation is necessary to clarify the role of the GLP-1 receptor itself (including postreceptor mechanisms like biased agonism) versus the endogenous GLP-1 peptide. This is especially critical given evidence across mice, rats, and humans that receptor agonism, but not increase of the endogenous peptide via dipeptidyl peptidase-4 (DPP-4) inhibition, is effective in SUD outcomes (21–23). The latter observation may be due, at least in part, to the overall superphysiological levels of GLP-1RAs and consequent level of receptor occupancy achieved with the newer long-acting GLP-1RA molecules, effects that are unlikely to be achieved with DPP-4 inhibition–related increases in endogenous GLP-1 levels. Shedding light on these mechanisms, including a better understanding of peripheral versus central actions and the extent to which the current generation of GLP-1RA molecules penetrate the blood-brain barrier, will be critical to better refine incretin-targeted therapies for SUDs and other medical conditions.

Finally, research on GLP-1–targeted therapies in SUDs and addictive behaviors has primarily focused on AUDs, OUDs, and TUDs with comparatively less attention given to stimulants, cannabis use disorder, and gambling. These areas represent important directions for future investigation, given the current limited pharmacological treatment options for this wide range of addiction-associated conditions. There has also been little focus on polysubstance use, even though polysubstance use disorders are more common than isolated SUDs (24), highlighting another critical gap in the literature. Future research should also investigate the optimal SUD treatment protocol with GLP-1RAs, including whether they are best utilized acutely in early recovery, as a long-term maintenance therapy, or both.

## Conclusions: where we started, where we are now

The development and final approval of GLP-1RAs for obesity was the culmination of decades of work spanning basic science to clinical translational efforts. Similarly, interest in GLP-1–targeted therapies for SUDs predates the more recent anecdotical reports. The hypothesis that GLP-1–targeted therapies could represent innovative pharmacological approaches for SUDs emerged more than a decade ago, and RCTs began well before the topic entered the public spotlight. The first preclinical studies on the effects of GLP-1–targeted therapies on alcohol, nicotine, and psychostimulant use came from a series of papers published in 2012–2013 from Aurelio Galli’s lab in the United States and from Elisabet Jerlhag’s lab in Sweden (for reviews from these labs, please see refs. 25, 26). A 2015 study from Lorenzo Leggio’s lab at the US NIH provided some of the earliest clinical evidence showing a genetically based link between GLP-1 receptor function and risk of AUD in humans (27). Since then, GLP-1–related preclinical and clinical research continues to grow exponentially, including EHR-based pharmacoepidemiology studies and RCTs. As is often the case when an early signal arises, enthusiasm outpaced evidence (28), but more recently we are observing encouraging positive results from well-controlled rigorous RCTs, as summarized above. While a firm evidence base of efficacy for use of GLP-1–targeted therapies for SUDs is not fully established, the results from recent RCTs represent a compelling step toward making these treatments available to patients. These studies, together with several ongoing trials of GLP-1–targeted therapies in SUDs across academia, the NIH, industry, and private sectors (see the several ongoing or recently completed RCTs registered in ClinicalTrials.gov) provide an encouraging outlook toward the potential of incretin-targeted therapies to open a new era in addiction science, addiction medicine, and, most importantly, for patients with alcohol and other SUDs.

## Conflict of interest

This work was supported in part by the Intramural Research Program of the NIH. LL reports, outside his federal employment, honoraria from the UK Medical Council on Alcohol (Editor-in-Chief for Alcohol and Alcoholism) and book royalties from Routledge (as editor of a textbook). WKS’s lab has received funding from Apollo Therapeutics and Altimmune Inc. as a clinical trial site in studies of GLP-1RA medications and has involvement in sponsored (preclinical) research agreements with Palatin Therapeutics. WKS is also listed as a coinventor on a provisional patent for weight loss (PCT/US2026/017411). WKS also currently serves on an advisory board for Eli Lilly Inc. and has formerly served on an advisory board for Apollo Therapeutics.

## Funding support

This work is the result of NIH funding, in whole or in part, and is subject to the NIH Public Access Policy. Through acceptance of this federal funding, the NIH has been given a right to make the work publicly available in PubMed Central.

- NIH intramural funding ZIA-DA000635 (Clinical Psychoneuroendocrinology and Neuropsychopharmacology Section; PI: LL), jointly supported by the National Institute on Drug Abuse Intramural Research Program and the National Institute on Alcohol Abuse and Alcoholism Division of Intramural Clinical and Biological Research.
- Hardesty Family Foundation grant to WKS.
- WKS also holds the Roger Hardesty Endowed Chair in Clinical Neuroscience at OSU.

## Version 1 09/01/2026

Electronic publication
